# Systemic Platelet-Activating Factor-Receptor Agonism Enhances Non-Melanoma Skin Cancer Growth

**DOI:** 10.3390/ijms19103109

**Published:** 2018-10-11

**Authors:** Eric Romer, Anita Thyagarajan, Smita Krishnamurthy, Christine M. Rapp, Langni Liu, Katherine Fahy, Azeezat Awoyemi, Ravi P. Sahu

**Affiliations:** 1 Department of Pharmacology and Toxicology, Boonshoft School of Medicine, Wright State University; Dayton, OH 45435, USA; eric.romer@wright.edu (E.R.); anita.thyagarajan@wright.edu (A.T.); christine.rapp@wright.edu (C.M.R.); liu.106@wright.edu (L.L.); fahy.3@wright.edu (K.F.); awoyemi.2@wright.edu (A.A.); 2 Department of Pathology and Dermatology, Boonshoft School of Medicine, Wright State University, Dayton, OH 45435, USA; smita.krishnamurthy@wright.edu

**Keywords:** PAF-R, cutaneous chemical carcinogenesis model, non-melanoma skin cancer

## Abstract

Platelet-activating factor-receptor (PAF-R) agonists are pleiotropic lipid factors that influence multiple biological processes, including the induction and resolution of inflammation as well as immunosuppression. PAF-R agonists have been shown to modulate tumorigenesis and/or tumor growth in various skin cancer models by suppressing either cutaneous inflammation and/or anti-tumoral adaptive immunity. We have previously shown that a chronic systemic PAF-R agonist administration of mice enhances the growth of subcutaneously implanted melanoma tumors. Conversely, chronic topical applications of a PAF-R agonist suppressed non-melanoma skin cancer (NMSC) in a topical chemical carcinogenesis model (dimethylbenz[a]anthracene/phorbol 12-myristate 13-acetate (DMBA/PMA)) in-part via anti-inflammatory effects. These results indicate that the context of PAF-R agonist exposure via either chronic cutaneous or systemic administration, result in seemingly disparate effects on tumor promotion. To further dissect the contextual role of PAF-R agonism on tumorigenesis, we chronically administered systemic PAF-R agonist, carbamoyl-PAF (CPAF) to mice under a cutaneous chemical carcinogenesis protocol, recently characterized to initiate both NMSC and melanocytic nevus formation that can progress to malignant melanoma. Our results showed that while systemic CPAF did not modulate melanocytic nevus formation, it enhanced the growth of NMSC tumors.

## 1. Introduction

Platelet-activating factor-receptor (PAF-R) is a widely expressed seven-transmembrane-spanning G protein-coupled receptor, which binds with varying affinities to platelet-activating factor (PAF, 1-0-alkyl-2-acetyl-sn-glycero-3-phosphocholine) or glycerophosphocholines that have been oxidatively modified on the sn-2 polyunsaturated fatty acid (oxGPCs) [1,2]. PAF and oxGPCs can be collectively referred to as PAF-R agonists. The stability of PAF-R agonists is regulated by PAF metabolizing enzymes, PAF-acetyl hydrolases (PAF-AH), which are found in multiple cell types and lipoproteins [3,4]. Studies, including ours, have shown that enzymatic (i.e., PAF) or non-enzymatically (e.g., ultraviolet B (UVB), cigarette smoke, jet fuel, and tumor promoters, such as PMA) generated PAF-R agonists mediate various pathophysiological effects, including enhanced growth of tumors in experimental models [5,6,7,8,9,10,11,12,13,14,15,16,17,18]. In particular, tumor growth promoting effects can be mimicked by the administration of a non-metabolizable form of PAF (1-hexadecyl-2-*N*-methyl carbamoyl glycerophosphocholine (CPAF)) [7,19].

Notably, the roles of PAF in experimental cutaneous carcinogenesis models remain complex. In particular, using PAF-R-expressing and -deficient cells or mouse models with a C57BL/6 background, we have shown that administration of systemic CPAF augments the growth of subcutaneously-implanted melanoma tumors [7]. However, the topical applications of CPAF suppress dimethylbenz[a]anthracene/phorbol 12-myristate 13-acetate (DMBA/PMA))-induced cutaneous non-melanoma skin cancer (NMSC) growth. The DMBA/PMA protocol is a dual chemical carcinogenesis model that induces papillomas and squamous cell carcinoma [7,19]. These divergent findings suggest that systemic PAF-R agonists may promote cutaneous tumors, while their topical applications may suppress them. Nevertheless, the effects of systemic PAF-R agonists on a cutaneous carcinogenesis model has not been tested. Notably, chronic systemic PAF-R agonist exposure is a physiologically and clinically relevant concern as the increase in systemic PAF-R agonists is associated with pathophysiological states including chronic infection, and xenobiotic exposure [9,10,11,19,20,21,22,23].

Given the intriguing roles of PAF-R agonists in modulating the growth of experimental melanoma and NMSC in response to diverse stimuli [7,15,19,23,24,25,26], the current study was designed to determine if systemic PAF-R agonist exposure modulates DMBA/PMA-induced cutaneous carcinogenesis. Our study took advantage of a recent report by Nasti et al., which characterized a DMBA/PMA cutaneous carcinogenesis model [27] in C3H/HeN mice that induces both NMSC and melanocytic nevi, which in turn can be transformed into malignant melanoma [28].

Our findings demonstrate that while systemic CPAF did not modulate DMBA/PMA-induced melanocytic nevus formation in C3H/HeN mice, it augmented the growth of NMSC tumors. Although, DMBA/PMA induced cutaneous carcinogenesis, particularly the initiation, multiplicity and growth of NMSC tumors appears to be uniform and unaffected by the mouse strains [12,19,28,29]. The ability of the PAF-R agonist via topical [19] and systemic (current study) applications seems to exert both tumor suppressive and tumor promoting activities on NMSC, respectively.

## 2. Results and Discussion

Several studies including ours have implicated the diverse roles of PAF-R agonists in modulating the growth of experimental tumor types in response to various stimuli [7,12,15,19,23,24,25,26]. In particular, our previous studies have shown that intraperitoneal injections of PAF-R agonist, CPAF, augment the growth of subcutaneously implanted murine melanoma tumors via mechanisms involving interleukin 10 (IL-10) and Tregs [7]. However, topical CPAF applications suppressed DMBA/PMA-mediated cutaneous tumorigenesis, and NMSC tumor growth via mechanisms partly associated with the suppression of chronic PMA-induced cutaneous inflammation and c-Kit+ mast cells [19]. Nevertheless, the effect of systemic CPAF on DMBA/PMA-induced cutaneous carcinogenesis has not been tested. 

**Figure 1:**
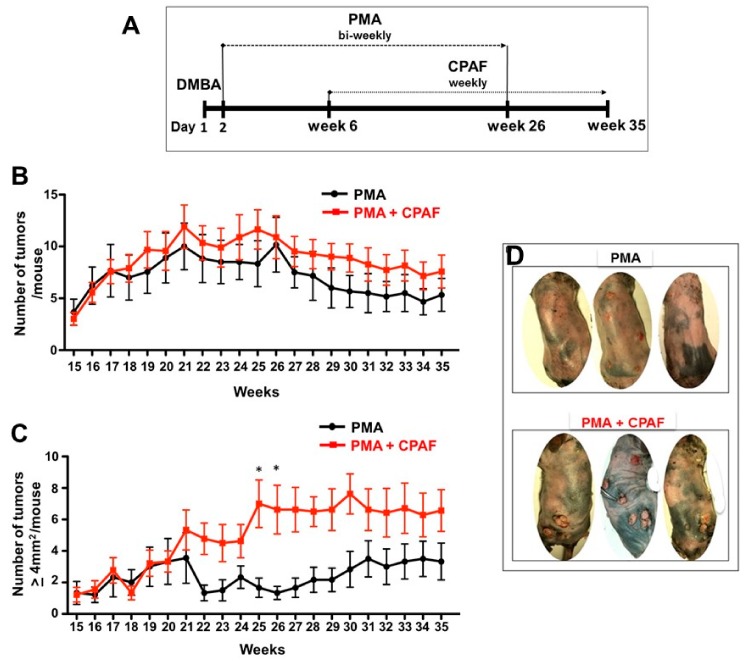
Effect of systemic CPAF on DMBA/PMA-induced tumor growth. (**A**) Schematic representation of our working model. Six weeks old C3H/NeH mice were treated topically with DMBA (100 µg/mouse for two consecutive days) followed by the treatments with PMA (50 µg/mouse; topically) with or without CPAF (250 ng/mouse; i.p.) for specific time points. (**B**) Total number of non-melanocytic tumors/mouse in PMA and PMA + CPAF groups is shown. (**C**) Total number of non-melanocytic tumors ≥4 mm^2^/mouse in PMA and PMA + CPAF groups is presented. (**D**) Representative photographs of mice from PMA and PMA + CPAF treated group is shown. * Represents statistical significance (*p* < 0.05) as determined by a 2-way ANOVA with Bonferroni post-hoc test.

In the current study, the murine chemical carcinogenesis model developed by Nasti et al. [28] was modified to determine the effects of chronic systemic PAF-R agonist exposure on the initiation and progression of cutaneous skin tumors. The shaved backs of C3H/HeN female mice were treated topically with a single dose of a tumor initiating carcinogen, (DMBA; 100 µg/mouse), and then treated bi-weekly with a tumor promoter (PMA; 12.5 µg/mouse). After an initiation period of 6 weeks, a group of mice were treated weekly with a systemic dose of CPAF (250 ng; i.p.) (Figure 1A). Starting at week 14, small epithelial tumors were observed on the dorsal skin of mice treated with PMA-alone and PMA + CPAF, but as expected, none were found in any of the non-PMA control animals.

The epithelial tumors continued to form and while some resolved, established tumors continued to grow over the next 10 weeks of treatment. By week 26, there was a non-significant trend for a greater number of tumors in the PMA + CPAF group as compared to the PMA-alone group (Figure 1B). When the tumors were analyzed by size, there were significantly larger tumors (a surface area of ≥4 mm^2^ when measured in two-dimensions) on the PMA + CPAF treated mice, compared to the PMA-alone controls (Figure 1C,D).

**Figure 2:**
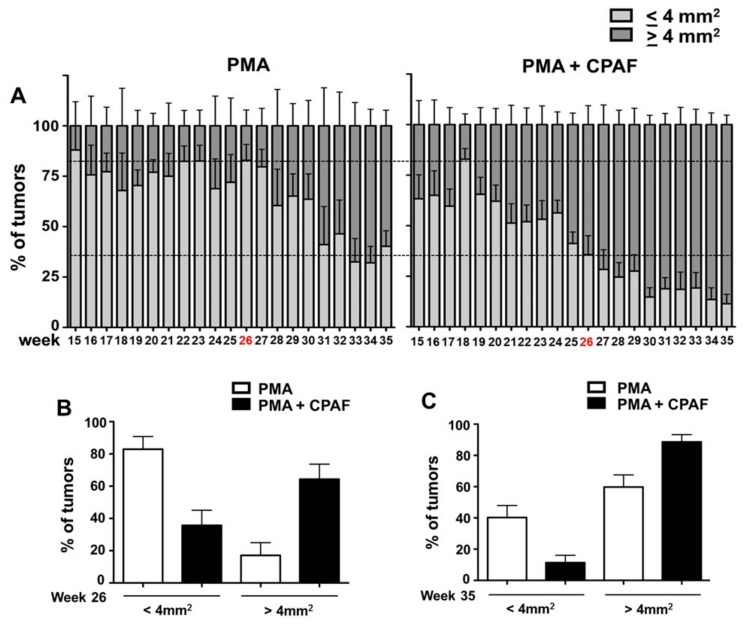
Effect of systemic CPAF on the invasiveness/durability of DMBA/PMA-induced tumors. (**A**) PMA treatment was stopped at week 26 (red color), and CPAF treatment continued for up to 35 weeks. Percentage of tumors of ≤4 mm^2^ (light gray color bar) or ≥4 mm^2^ (dark gray color bar) in PMA and PMA + CPAF treated groups of mice are shown. (**B**,**C**) Percentage of all tumors in PMA and PMA + CPAF groups at week 26 and 35 are shown.

At week 26, 64% of the tumors were ≥4 mm^2^ on the PMA + CPAF treated mice, while only 17% of the PMA-alone tumors met the size threshold (Figure 1D and Figure 2A,B). There was a greater number of larger tumors (i.e. ≥4 mm^2^) on the PMA + CPAF treated mice (Figure 1D and Figure 2A,B). To test the durability of these epithelial tumors with PAF-R agonist exposure, the bi-weekly tumor promoting PMA treatments were ceased but the animals continued to receive the weekly systemic treatments with CPAF for approximately 10 more weeks. By week 35, the small tumors (≤4 mm^2^) which made up a percentage of all tumors, decreased in the PMA-alone (40% of all tumors) and in the PMA + CPAF (11% of all tumors) mice during this period (Figure 2C).

In addition, the mean two-dimensional area of those larger tumors was significantly different from the PMA treated group at 35 weeks (Supplemental Figure S1A). Interestingly, significantly more PMA + CPAF epithelial tumors grew ≥2 mm up from the surface of the skin than the tumors of the PMA alone controls (Supplemental Figure S1B). These findings demonstrate that chronic systemic PAF-R agonist exposure promotes DMBA/PMA-induced cutaneous NMSC tumor growth.

While a trend for a greater number of larger tumors in the PMA + CPAF group persisted at 35 weeks, the difference was no longer statistically significant (Supplemental Figure S1B). The two-dimensional size and height of the larger PMA + CPAF tumors significantly increased between weeks 26 and 35 (Supplemental Figure S1A,C), but there were no statistically significant changes in these parameters in the PMA-alone tumors. These data collectively suggest that as expected, the cessation of PMA treatment suppressed new tumor formation, and the continued treatment with systemic CPAF promoted the growth of persisting tumors.

It is important to note that in this study, we did not notice any significant differences in the multiplicity or incidence rate of all tumors, suggesting that the formation of new tumors was not impacted by systemic CPAF, whereas topical CPAF appeared to decrease the formation of new tumors [19]. Therefore, a chronic concentration of topical CPAF on the skin may cause an anti-inflammatory milieu that suppresses PMA-mediated tumor promotion and subsequently the formation of new cutaneous tumors [19].

These studies are consistent with the previous report demonstrating that topical applications of PMA resulted in significantly greater numbers of skin tumors in CD-1 mice compared to non-PMA treated groups [29]. Although they are not directly related to the increased growth of non-melanoma skin tumors, studies including ours have shown that CPAF treatments increase the in vitro proliferation of melanoma and non-melanoma tumor cells in a PAF-R-dependent manner [24,30].

As previously shown by Nasti et al. [28], nevi also formed on the mice that received DMBA and PMA, but systemic CPAF administrations did not increase or decrease the formation of nevi throughout the experiment (Supplemental Figure S2). The fact that systemic CPAF does not appear to modulate the formation of PMA-induced melanocytic nevi in this experimental mouse model, could be because CPAF may exert distinct effects on melanocyte biology and NMSC via a complex interplay between multiple cell types with counter-regulatory functions. Notably, increased pigmentation (at 46 weeks) as well as the number of melanocytic tumors (at 28 months) have been noted on the skin of PAF-R transgenic mice compared to the normal control mice [31]. These findings indicate that PAF-R overexpression upon aging may contribute to melanomagenesis, however, the molecular mechanism of this event remains unclear. Nevertheless, if topical CPAF applications could accelerate the PMA-induced formation of melanocytic nevi, it would be an interesting area of investigation for future studies.

In summary, this study demonstrates that systemic CPAF treatment enhances the growth of non-melanocytic tumors induced by a repetitive DMBA/PMA carcinogenesis model in C3H/NeH mice. As various immunophenotypes, non-immune cells and factors are modulated in response to PAF-induced tumorigenesis [7,12,15,19,23,24,25,26], future studies are warranted to determine the detailed molecular mechanism(s) of systemic CPAF-mediated effects in this experimental cutaneous carcinogenesis model, and topical CPAF effects on the subcutaneous melanoma model. In addition, whether or not C3H/NeH mice crossed with PAF-R deficient mice with a C57BL/6 background will exhibit distinct tumor modulating activities under the DMBA/PMA protocol. This is a goal for future studies.

## 3. Materials and Methods

### 3.1. Reagents and Chemicals

Phorbol 12-myristate 13-acetate (PMA) was purchased from Promega, (Madison, WI) and 7,12-Dimethylbenz(a)anthracene (DMBA) from Acros Organics, (Fair Lawn, NJ, USA). Carbamoyl-PAF was obtained from Sigma-Aldrich (St. Louis, MO, USA). All other reagents were purchased from ThermoFisher Scientific, (Waltham, MA, USA).

### 3.2. Mice

C3H/NeH mice at the age of 6 weeks were purchased from Charles River Laboratories and housed in pathogen free conditions. The animal procedures and protocols (AUP# 1075) were approved on October 18th, 2016 by the institutional animal care and use committee (IACUC) at Wright State University (WSU).

### 3.3. Experimental Chemical Carcinogenesis Model and Systemic CPAF Application

These mice (6–8 per experimental group) were anesthetized with ketamine/xylazine and paired to remove the dorsal hair at the start, and before each treatment and measurement of tumors. Dorsal skin was topically painted with 100 µg of DMBA on day 1, followed by 50 µg of PMA in 200 µL of vehicle (3:1 ratio of acetone and olive oil) which started on day 2 and was repeated twice weekly until week 26 as shown in Figure 1A. Treatment with intraperitoneal injections of CPAF at 250 ng/100 µL doses started at the beginning of week 6 and was repeated once every week until week 35. Control mice were treated topically with the vehicle (200 µL solution of acetone/olive oil) alone. These mice were monitored closely twice a week and the formation of melanocytic nevi and non-melanocytic tumors were recorded by counting their numbers. The two-dimensional surface area of tumors was determined by measuring in two directions (length and width). The products measuring ≥4 mm^2^ are described as larger tumors, while the tumors that were ≤4 mm^2^ are described as smaller tumors. At week 26 and 35 the heights of tumors ≥2 mm^2^ were measured by digital caliper and presented as an average height of tumors. 

### 3.4. Statistical Analysis

At least 6–8 mice were used in these studies. Statistical significance was assessed by Prism 5.0 software (Graph Pad Software, San Diego, CA, USA) using 2-way ANOVA with Bonferroni post-hoc test. The significance was set as *p* < 0.05.

## Supplementary Materials

Supplementary materials can be found at http://www.mdpi.com/1422-0067/19/10/3109/s1.

## Abbreviations

PAF	Platelet-activating factor
PAF-R	Platelet-activating factor-receptor
CPAF	1-hexadecyl-2-*N*-methyl carbamoyl glycerophosphocholine
DMBA	Dimethylbenz[a]anthracene
PMA	Phorbol 12-myristate 13-acetate
NMSC	Non-melanoma skin cancer
