# Supplementary material for: Systemic Platelet-Activating Factor-Receptor Agonism Enhances Non-Melanoma Skin Cancer Growth
Source: Int J Mol Sci. 2018 Oct 11;19(10):3109. doi: 10.3390/ijms19103109 (PMC6212876; doi:10.3390/ijms19103109)
Supplement: Supplementary file 1 [file ijms-19-03109-s001.pdf]

Supplementary Figure 1

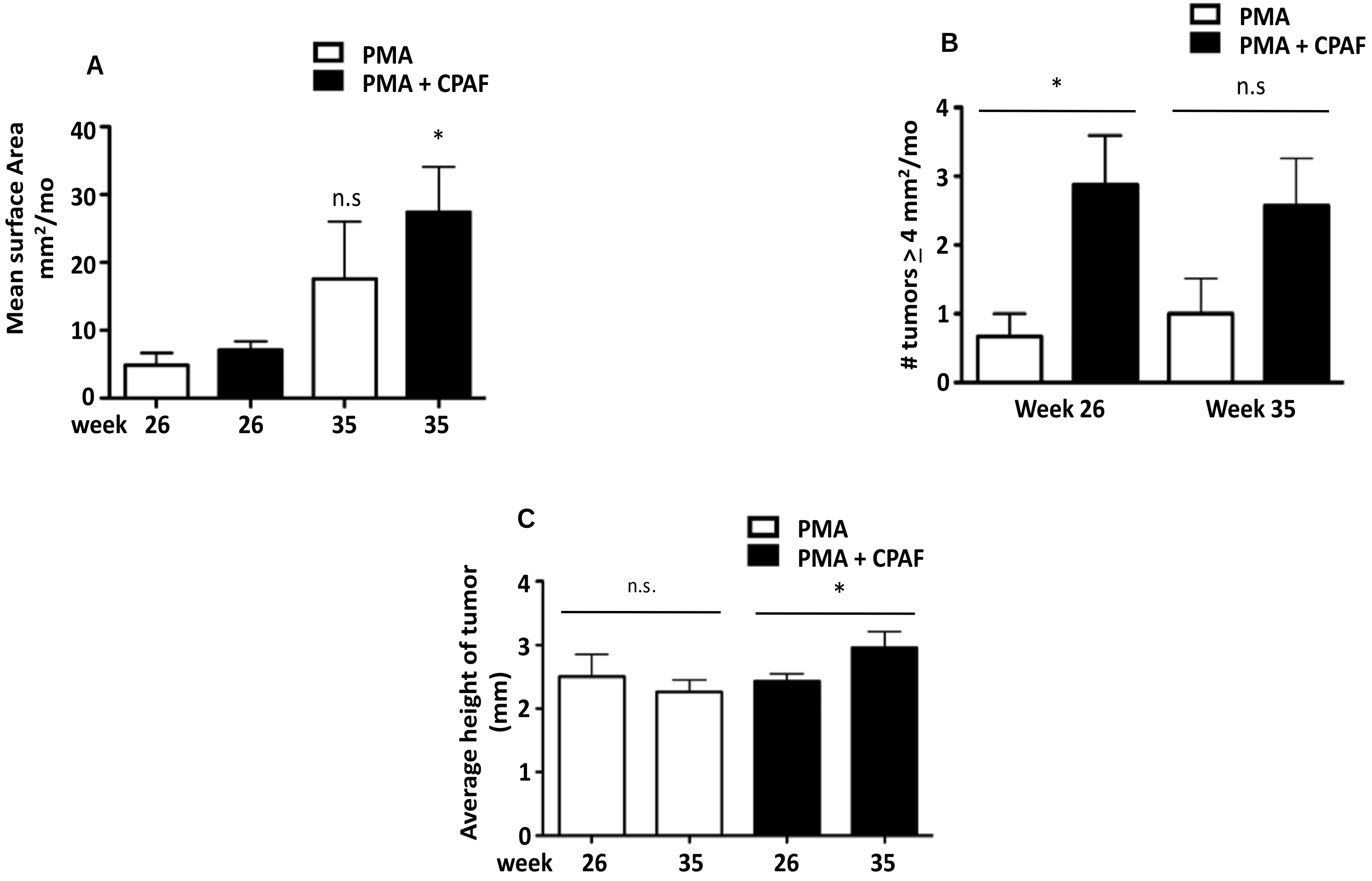

Supplemental Figure 1. Systemic CPAF increases the growth of tumors. A) The mean surface area (mm^2/mouse) in PMA (white) and PMA+CPAF (black) at 26 and 35 weeks are shown. *denotes statistical significance relative to 26 week PMA+CPAF by two-tailed student t-test ($p<0.05$). **B-C)** The average height of tumor (mm) or tumors of ≥ 2 mm height/mouse in PMA (white) and PMA+CPAF (black) groups at 26 and 35 weeks are shown. *denotes statistical significance by two-tailed student t-test ($p<0.05$).

Supplementary Figure 2

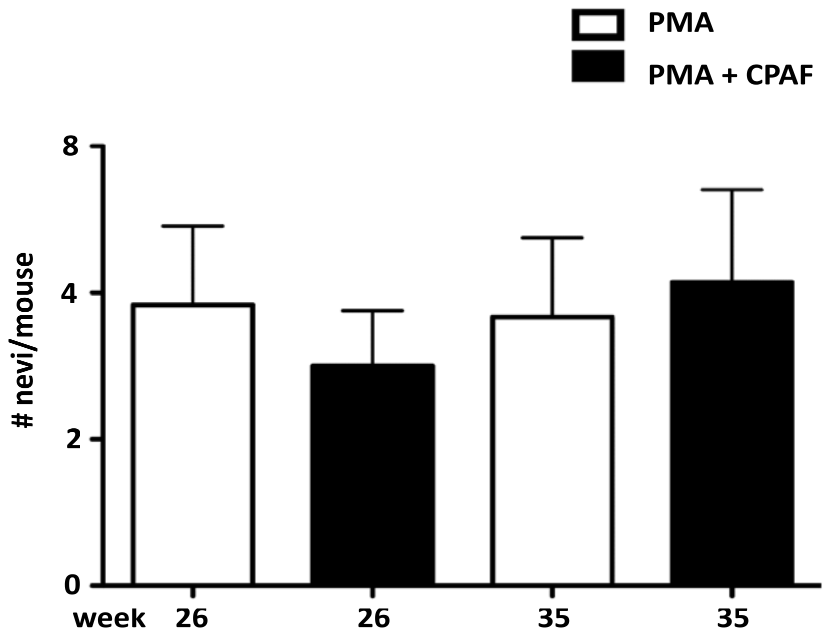

Supplemental Figure 2. Systemic CPAF does not modulate the number of melanocytic nevi. The number of melanocytic nevi was counted at every week. The data represent as number of nevi/mouse at week 26 and 35.
